# High-Temperature Hydrogen Permeability Tests of Ta Tubes

**DOI:** 10.3390/membranes16070219

**Published:** 2026-06-26

**Authors:** Damiano Capobianco, Silvia Zanlucchi, Teresa Beone, Lorenzo Bartolucci, Stefano Cordiner, Gessica Cortese, Luca Farina, Vincenzo Mulone, Egidio Zanin, Silvano Tosti

**Affiliations:** 1 RINA Consulting-Centro Sviluppo Materiali S.p.A., 00128 Roma, Italy; damiano.capobianco@rina.org (D.C.); silvia.zanlucchi@rina.org (S.Z.); teresa.beone@rina.org (T.B.); egidio.zanin@rina.org (E.Z.); 2 Department of Industrial Engineering, Tor Vergata University of Rome, Via del Politecnico, 1, 00133 Rome, Italy; lorenzo.bartolucci@uniroma2.it (L.B.); stefano.cordiner@uniroma2.it (S.C.); vincenzo.mulone@uniroma2.it (V.M.); 3 Nuclear Department, ENEA, C.R. Frascati, Via Enrico Fermi, 45, 00044 Frascati, Italy; gessica.cortese@enea.it

**Keywords:** hydrogen separation, hydrogen permeability, tantalum membrane tubes

## Abstract

Refractory metals are being studied as alternatives to Pd and its alloys for the separation of hydrogen in high-temperature processes. The development of a membrane reactor for the production of hydrogen via water splitting has required studying hydrogen permeability through Ta at temperatures above 1273 K, for which no data is available in the literature. A dedicated experimental setup has been realized for testing Ta tubes in the temperature range 673–1573 K. Despite the use of controlled atmospheres and ultra-pure gases (with oxygen content below a few ppm), the tests over 1473 K have involved the formation of oxide layers over the metal surfaces, as verified by SEM-EDS analyses. The presence of oxide layers significantly increases the energy barrier to permeation: in agreement with a modest surface oxidation, at lower temperatures (673–1273 K) the activation energy of 2679.8 K has been measured against the value of 30,691 K measured in the high-temperature tests (1473–1573 K).

## 1. Introduction

The world’s need of energy is continuously growing and, in parallel, energy security plays a key role in the resilience of economic and industrial systems. In 2025, renewables deployment sets new records following a positive trend over the last 20 years, while electricity demand rose faster than overall energy use [1]. Despite the penetration of renewable energy, global energy-related CO_2_ emissions continued their evolution and reached 38 Gt, making it inevitable that the global warming target of 1.5 °C temperature increase will be exceeded [1]. Strategic foresight analyses consider global warming as a main impacting megatrend for the near future, capable of threatening humanity’s sustainable development [1,2]. As a clean energy vector, hydrogen has great potential to contribute to the efficient and sustainable exploitation of renewables. In 2024, the global hydrogen demand achieved about 100 Mt with a growth rate of 25 in line with that of the overall energy demand [1,3,4]. However, hydrogen production is not free from environmental impacts, and its pollution level has been categorized by introducing a “color gradation”: green hydrogen is produced through the electrolysis of water powered by renewable sources, while hydrogen production correlated with CO_2_ emissions is assigned various other colors [5,6,7]. In practice, a series of colors from green to black indicates hydrogen production processes characterized by no direct CO_2_ emissions to very high levels of emissions [5].

Today, the hydrogen on the market has a high associated carbon footprint since it is produced by about 96% from fossil fuels and, particularly, 49.5% from natural gas, 29% from liquid hydrocarbons and 18% from coal [5,8]. Difficulties in producing low-emissions hydrogen include high costs, uncertain demand and regulations, and slow infrastructure development [1,3,9]. Grey hydrogen produced via steam natural gas reforming exhibits the lowest cost (1.5–2.5 $/kg), while the blue hydrogen produced via steam natural gas reforming with CO_2_ capture is around 2.00–2.50 $/kg [10]. Green hydrogen, which is presently estimated at 3.50–6.00 $/kg, could benefit from tax credit and future technology improvements [10]. In China, an assessment has foreseen that the cost of hydrogen produced by PV-assisted alkaline electrolysis will move from 4.43 to 3.63 $/kg from 2025 to 2035 [11]. In a study considering several sites around the world, the LCOH of PV-PEM systems is expected to reach a minimum of 5.6 $/kg [12], while, when PV is combined with wind turbines, the LCOH reduces to 2.92 $/kg [13].

Further, the production of “green” hydrogen via water electrolysis powered by electricity produced by PV panels is one of the promising technologies currently under study for both storing and exploiting solar energy: the hydrogen produced in this way can be converted into electricity according to the grid demands or used to produce fuels and chemicals in power-to-fuel processes [14,15,16].

A process under consideration to reduce the cost of green hydrogen foresees the direct use of solar energy to power a high-temperature water-splitting reactor [17]. Although it poses severe technological challenges due to its operation at very high temperatures, such a process avoids the installation of extensive PV and electrolyzer systems, thereby reducing the CAPEX and OPEX costs of hydrogen production plants. The water-splitting reaction is promoted by high temperatures and low pressures [18]:

H_2_O ⇄ H_2_ + ½ O_2_   ΔH_298 K_ = 241.8 kJ mol^−1^ (1)

At atmospheric pressure, for achieving a reaction conversion of 10%, it is necessary to operate over 2773 K. Such operating conditions make the direct use of solar energy for powering the water splitting unfeasible, and the research interests have been focused on processes based on complex thermochemical cycles, which have been studied [19,20]. Alternatively, when a membrane capable of selectively removing one or more reaction products is introduced into the water-splitting reactor unit, the reaction conversion can exceed the thermodynamic limit. For instance, a water-splitting reactor with a hydrogen-permeable membrane has been studied by Sui et al. [21], who reported high conversion rates at 1773 K and very low pressure (1 Pa). Further, a membrane reactor consisting of two selective membranes for extracting hydrogen and oxygen separately has been designed: in this process unit, the synergistic action of the two distinct membranes allows shifting reaction (1) well beyond thermodynamic equilibrium. At 50 kPa, the reaction conversion of 20% and 10% has been preliminarily estimated for a double-membrane solar reactor (with membrane efficiency 90%) at around 2273 K and 2073 K, respectively, while a traditional reactor exhibits the same reaction conversions (20 and 10%) at around 3073 and 2773 K [17].

Refractory body-centered (bcc) metals exhibit higher hydrogen permeability than Pd and its alloys [22]. Even these metals are highly reactive with oxygen and other gases; they can operate at very high temperatures (over 2273 K) and exhibit high hydrogen permeability. Based on these considerations, in this work tantalum tubular membranes have been considered for hydrogen separation.

The hydrogen mass transport through a dense metal wall takes place with a series of steps:

- Adsorption and decomposition of hydrogen molecules into atoms over the metal surface upstream.
- Diffusion of the hydrogen atoms through the metal lattice.
- Desorption of the hydrogen atoms and their combination into molecules over the downstream metal surface.

The permeability is a lumped parameter expressing the hydrogen transport through metals. Under the diffusion-controlled regime, the permeation phenomenon is ruled by the Sieverts’ law in which the hydrogen permeated flow rate is proportional to the difference of the square root of the hydrogen partial pressures:

(2) $$F=Pe\frac{\sqrt{p_{up}}-\sqrt{p_{down}}}{th}A$$

where *F* is the hydrogen flow rate (mol s^−1^), *Pe* is the permeability coefficient (mol m^−1^ s^−1^ Pa^−0.5^), *A* is the surface area of the tubular membrane (m^2^), *th* is the thickness of the membrane (m), and *p_up_* and *p_down_* are the partial pressure of hydrogen upstream and downstream (Pa), respectively.

In the presence of oxidized layers covering the metal, the surface reactions (dissociation/recombination and adsorption/desorption of hydrogen) may control the hydrogen mass transfer and in Formula (2) the exponent of the partial pressures move from 0.5 to 1.

Since permeability is an activated process characterized by an activation energy coefficient, its dependence on temperature is ruled by an Arrhenius law:

(3) $$Pe={Pe}_{0}e^{-\frac{E_{a}}{T}}$$

where: ${Pe}_{0}$ is the pre-exponential factor (mol m^−1^ s^−1^ Pa^−0.5^), *E_a_* is the activation energy (K) and *T* (K) is the temperature.

Few data of Ta permeability measurements are reported in the literature. In fact, the formation of passive oxide layers can significantly reduce the transport of the hydrogen through the metal membranes, thus making the results of the experiments difficult to reproduce. Mostly, the permeability of hydrogen through Ta has been indirectly calculated by the product of the distinct measured values of solubility and diffusivity of hydrogen in the metal. Rothenberger et al. [23] reviewed the literature data available for the measured hydrogen transport properties of Ta and propose in the temperature range 948–1073 K the values of the pre-exponential coefficient and energy activation reported equal to 1.0 × 10^−6^ mol m^−1^ s^−1^ Pa^−0.5^ and 3240 K, respectively. It is noteworthy that such permeability values have been obtained from solubility measurements carried out from 625 to 944 K and diffusivity measurements in the temperature range of 948–1073 K. Besides the approximation introduced by coupling experimental data (diffusivity and solubility) obtained in different temperature ranges, the values of permeability assessed in this way cannot account for the presence of oxidized Ta surfaces.

The development of a project to design a double-membrane water-splitting reactor powered by solar energy should require:

- Availability of permeability data of hydrogen through Ta at temperatures above 1073 K.
- Measurements of permeability carried out on passive metal surfaces representative of the reaction environment where some percent of oxygen is present.

Accordingly, this work reports the results of experiments dedicated to the measurement of hydrogen permeability through Ta up to 1573 K.

## 2. Experimental Section

### 2.1. Material and Methods

To study the hydrogen permeation through Ta at high temperatures, an optimal experimental setup was developed. The membranes tested in the experimental campaign consist of commercial Tantalum (Ta bcc crystal structure) closed-ended tubes, also known as a finger-like configuration, purchased from WHS Sondermetalle Gmbh (Deining, Germany), having an outer diameter of 25.4 mm, a wall thickness of 0.4 mm and a length equal to 280 mm.

#### 2.1.1. Experimental Setup

**Figure 1:**
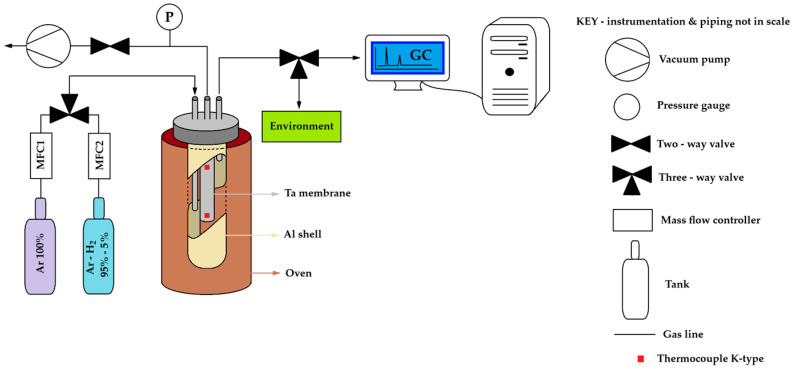
Sketch of the instrumentation and piping adopted for the hydrogen permeation in the Ta membrane.

Figure 1 shows the experimental setup. The Ta membrane with a welded sleeve is placed inside an alumina shell with an AISI 316 flange and is provided with inlet and outlet lines of sweep gas (Ar or Ar-H_2_) on the shell side.

**Figure 2:**
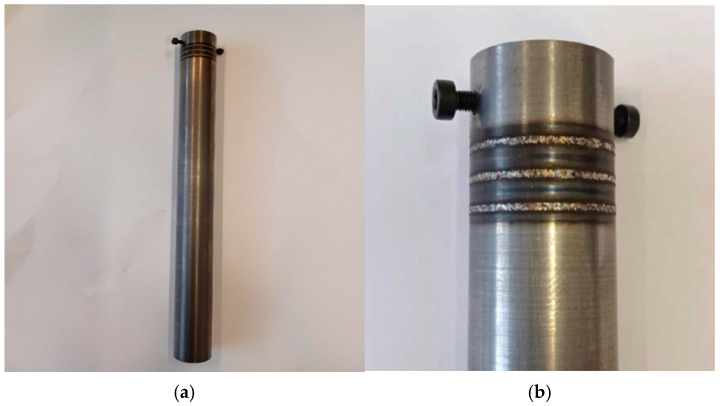
(**a**) Ta membrane coupled to the stainless-steel sleeve; (**b**) picture of the welding between the Ta membrane and the stainless-steel sleeve.

The tight sealing of the Ta tube to the module shell has been realized through the joining of Ta to a stainless-steel sleeve via the laser welding apparatus nLight Corona CFX-5000. After welding tests performed on several specimens of a different Ta tube, the following operating parameters of the laser apparatus were adopted: power in the range 350–2500 W, speed up to 33 mm s^−1^, spot diameter 0.1–0.3 mm. Figure 2a,b show the membrane after the welding process: in particular, three parallel welding seams have been realized in order to increase the reliability of the joining. Finally, the Ta tube involved in the experimental campaign has been joined via laser welding to a stainless-steel sleeve that, in turn, has been welded to an AISI 316 flange coupled to an alumina shell. Before starting the experimental campaign, several tests were performed on a different Ta tube to optimize the weldings between the Ta tube and stainless-steel sleeve.

After the insertion of the joining sleeve, the Ta tube has a useful length of 230 mm and a diameter of 24.5 mm. The total volume of the membrane is 0.00013 m^3^.

Two thermocouples located at the inlet and at the bottom of the membrane measure the actual temperature in the permeation zone. The shell is located inside a heating system provided by an oven with electric resistances and a thermocouple for temperature monitoring. A vacuum pump (Alcatel) is connected to the membrane to evacuate the hydrogen that permeates during oven ignition. The pressure gauge (smc ZSE40) installed downstream of the vacuum pump measures the pressure change inside the membrane due to hydrogen permeation. A line for the Gas Chromatographer (Agilent Micro GC 990) analysis was used to measure the hydrogen concentration inside the shell during the permeation tests and to verify the actual percentage of H_2_ outside the membrane (shell side).

#### 2.1.2. Permeation Tests

This section describes the experimental procedure aimed at measuring the high-temperature hydrogen permeability coefficients through a Ta tubular membrane. Before and after each permeation test, the membrane’s integrity and the absence of leaks in the experimental setup were checked at room temperature. In the leak tests, the membrane lumen was filled with inert gas (Ar) by pressurizing the system to about 300 kPa and verifying that the pressure was maintained for 1500 s. During the permeability tests at different temperatures (673–1573 K), the absence of leaks was demonstrated by flowing Ar in the membrane ramp-up, then keeping the membrane lumen vacuum-pumped until the test temperature was achieved and verifying no increase in the pressure in the membrane lumen. After this leak check, the Ar/H_2_ mixture was fed for performing the permeation test.

Single permeability measurements have been carried out at each test temperature to evaluate the permeation properties according to the following procedure:

- The membrane lumen is vacuum-pumped (≃200 Pa) until the achievement of the test temperature.
- The heating begins with a ramp rate of 673 K h^−1^.
- Starting from time t_0_ when the system is at room temperature, Ar is sent into the shell to protect the membrane and the steel sleeve from any oxidation due to the presence of air.
- Once the test temperature is achieved, the valve between the membrane lumen and the vacuum pump is closed and 100 NL h^−1^ of Ar-H_2_ (Ar 95 vol%, H_2_ vol5%, O_2_ 0.5–3 ppm) is sent into the shell.

The pressure increase in the membrane lumen vs. time has been measured by the manometer, and, in parallel, gas chromatography has verified the hydrogen concentration on the shell side. The test temperature has been varied in the range 673–1573 K by executing three testing cycles:

- A first cycle at 673, 873 and 1073 K.
- A second cycle at 1173, 1273, 1473 and 1573 K.
- A third cycle holding the system at 1473 K.

**Figure 3:**
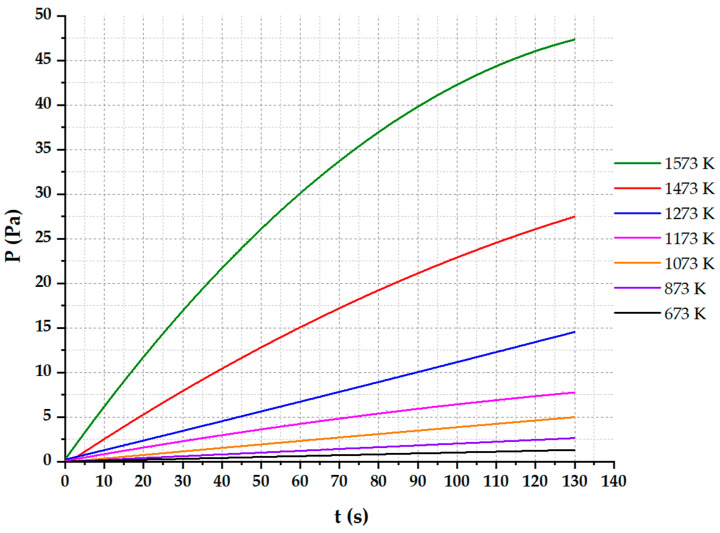
Behaviour of the pressure in the membrane lumen as a function of the time at 673 K (black), 873 K (purple), 1073 K (orange), 1173 K (magenta), 1273 K (blue), 1473 K (red), and 1573 K (green).

The behavior of the pressure measured by the vacuum gauge in the membrane lumen is shown in Figure 3.

According to the permeation model presented in the following section, the pressure variation measured in the membrane lumen is proportional to the amount of hydrogen permeated and increases with the temperature.

### 2.2. Permeation Model

For the permeation tests carried out in dead-flow mode, analysis of hydrogen permeation through the Ta membrane tube yields a relationship between the hydrogen pressure in the membrane lumen and the permeability coefficient, once the system geometry is known [24].

The hydrogen flow rate permeated is related to the variation in the number of moles (*n*) inside the lumen of the tubular membrane in the unit of time *t*(s) expressed by the following equation:

(4) $$F=\;\frac{dn}{dt}$$

According to the ideal gas law:

(5) P V=n R T

where *V* is the total volume of the membrane (m^3^) and *R* corresponds to the universal gas constant (8.314 J mol^−1^ K^−1^).

Equations (4) and (5) can be combined into expression (2), presenting the relationship of hydrogen permeation flow rate and hydrogen partial pressure. Deviations from Sieverts’ law can occur in the presence of oxidized surfaces and, accordingly, the permeability values obtained in this experimental campaign will be commented on in the following Sections.

The upstream pressure ($p_{up}$) is constant and equal to the partial pressure of hydrogen in the scrubbing gas (5% of 1 bar) and therefore results in:

(6) $$\sqrt{p_{up}}=\;k\;=\;\sqrt{5000}\;{\mathrm{Pa}}^{0.5}$$

Combining Formulas (2) and (4)–(6), the following expression can be obtained:

(7) $$\frac{dP}{dt}\frac{V}{R\;T}=\;\frac{Pe}{th}\;A\;\left(k-\;\sqrt{p_{down}}\right)$$

The downstream partial pressure of hydrogen (*p_down_*) is measured in the experimental apparatus as the temperature varies and constitutes the variable *P* = *f(t)* in the following differential equation:

(8) $$\frac{dP}{k-\;\sqrt{P}}=\;\frac{Pe\;A\;R\;T}{th\;V}\;dt$$

where $t_{in}$ = 0 and $t_{fin}$ = 120 s correspond to the values${\;P}_{in}$ = 0 Pa and $P_{fin}$ obtained from the experiments and are solved with respect to *Pe* leading to:

(9) $$Pe=\;\frac{-2\;\sqrt{Pfin}\;-\;2k\ln\left|\;k-\;\sqrt{Pfin}\;\right|+\;2k\ln\left|k\right|}{A\;R\;T{\;t}_{fin}}\;V\;th$$

### 2.3. Assessment of the Permeability Coefficient

**Figure 4:**
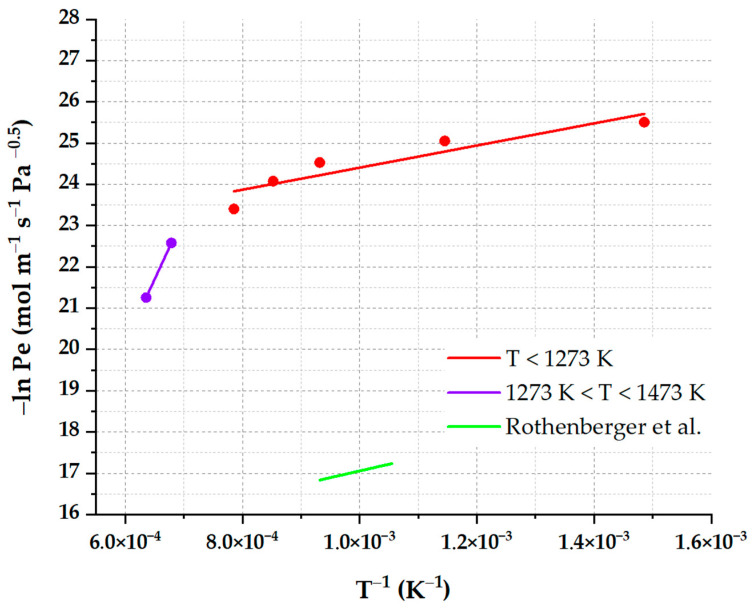
Comparison of hydrogen permeability obtained in experimental tests and literature [23].

The values of hydrogen permeability calculated via expression (9) are reported in the graph of Figure 4.

The results of the permeation tests suggest different behavior of the material in measurements carried out up to 1273 K and in the range 1473–1573 K. For the corresponding temperature ranges, the linear regression analysis provided the pre-exponential factors and the activation energy reported in Table 1.

Using the values of the pre-exponential coefficient and activation energy proposed by Rothenberger et al. for the temperature range 948–1073 K (see Table 1), the hydrogen permeability through Ta is shown in Figure 4 and compared with the results obtained in the experimental tests.

## 3. Results and Discussion

The different behavior of the Ta observed at low and high temperatures can be related to the state of the membrane tube surfaces after the tests.

### 3.1. Tantalum Oxidation

**Figure 5:**
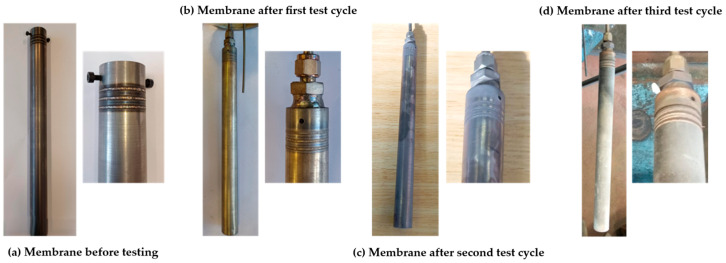
(**a**–**d**) View of the membrane surface after each test cycle.

Figure 5a–d show the Ta membrane tube before testing and after each of the three test cycles.

From Figure 5a–d, it is evident that the higher the test temperature, the greater the oxidation of the membrane surface. In fact, during the second and third test cycles carried out in the range, the membrane surface shows an increasing formation of oxide layers.

The oxidation of Tantalum has been reported in the literature by several authors. At lower temperatures (323–573 K), the oxidation proceeds with only minor recrystallisation of the films [25], while between 673 and 803 K, the continuous growth of the oxide layers involves a volume expansion responsible for their cracking [26]. In general, the oxides consist of Ta_2_O_5_, with TaO representing the intermediate product at the metal/oxide interface below 1073 K [27]. At higher temperatures (1573–1773 K), the presence of atomic oxygen has been observed to significantly increase the extent of oxidation [28].

**Figure 6:**
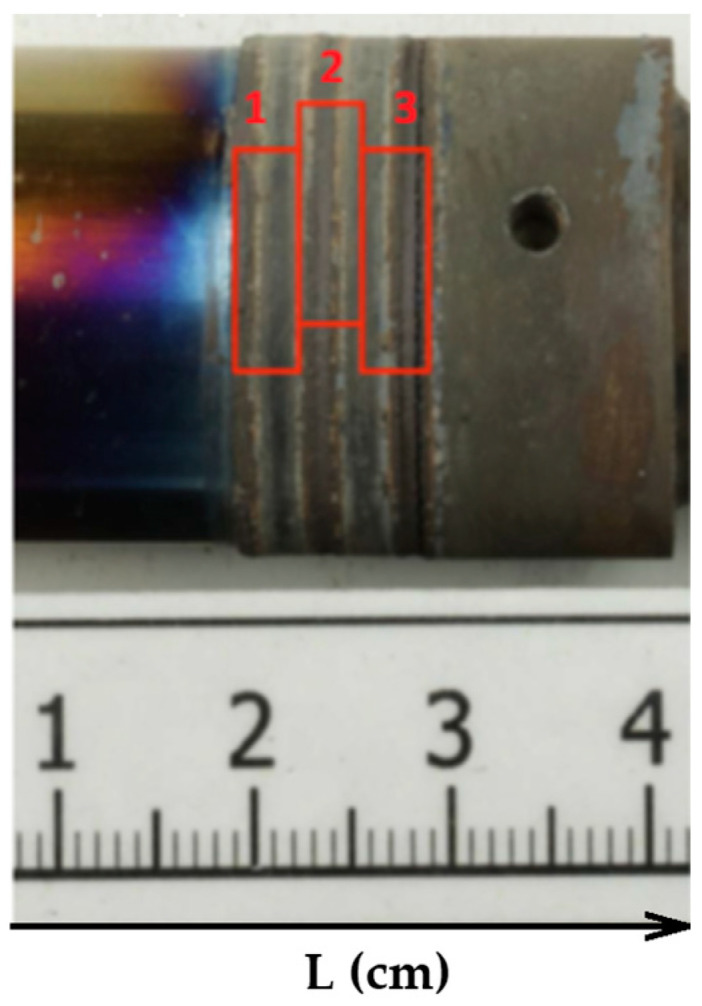
Selected sites (1, 2, 3—red rectangles) for SEM analysis of the joining after permeation tests.

**Figure 7:**
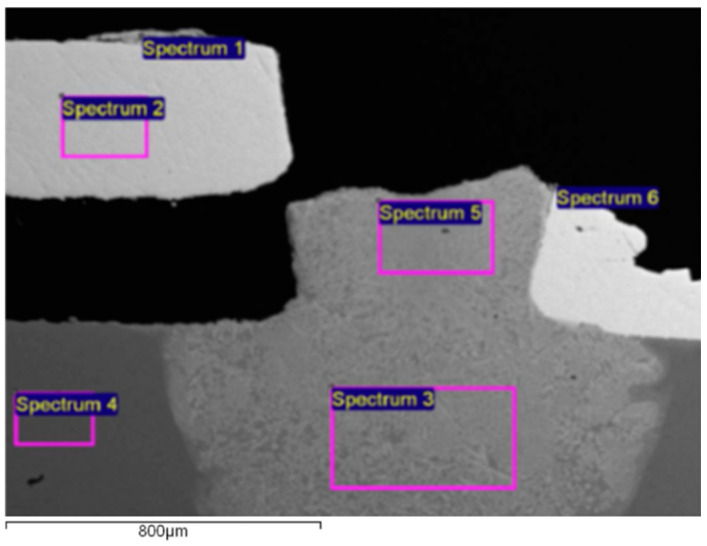
SEM image of the cross-section in site 2, coupled with the EDS analysis (pink rectangle).

In the experimental campaign, the presence of oxygen in the Ar-H_2_ gas mixture (namely at a level of 0.5–3 ppm) is therefore sufficient to cause surface oxidation in the tests at high temperature, particularly when operating at 1473 K. These hypotheses have been verified by EDS analysis of the membrane surface. Before testing, the metal surface has an oxygen concentration of about 4–5% or below, while after testing, it rises to over 8% (weight percentage–wt. %). As an example, Figure 6 and Figure 7 show the Ta surface in correspondence with its joining with the stainless-steel sleeve and the SEM image of site 2, whose EDS analysis is reported in Table 2. In particular, “spectrum 1” and “spectrum 2” relate to the Ta surface exposed and non-exposed to the gas phase, respectively. Accordingly, “spectrum 1” reports the presence of oxygen at 8.84%.

**Figure 8:**
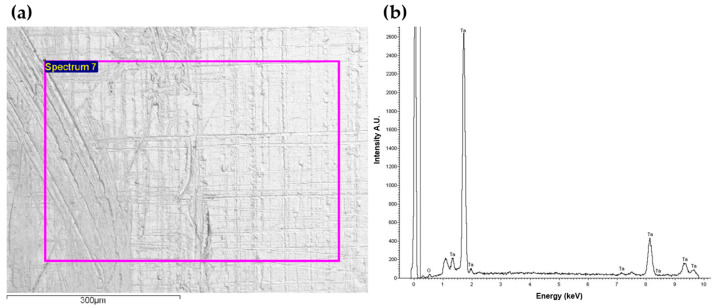
(**a**,**b**) SEM-EDS of the quasi-pristine Ta surface sample.

**Figure 9:**
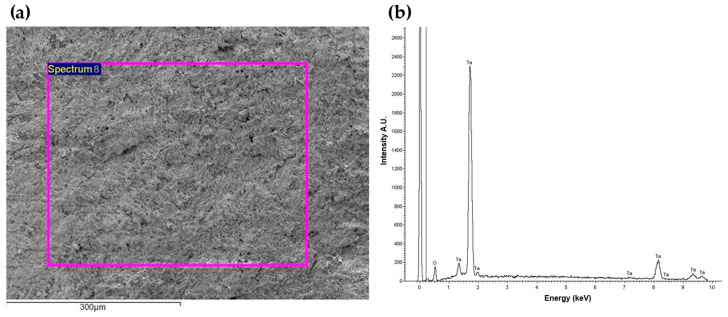
(**a**,**b**) SEM-EDS of the Ta after the permeation tests performed at the higher temperature.

For evaluating more in depth the chemical properties of the metal surfaces, Figure 8a,b and Figure 9a,b show a comparison of the SEM-EDS investigation carried out before (quasi pristine metal) and after the permeation test at the higher temperature. In particular, the investigation of the samples after the permeation tests concerned surfaces far from the weldings that could have further affected the Ta tube surface state.

Finally, Table 3 summarizes the EDS quantitative analysis carried out of the samples pre- and post-thermal cycling, outlining the increase in the oxygen weight percentage from 1.74% to 8.03%.

### 3.2. Analysis of Results and Comparison with Literature

The Ta membrane tested has exhibited permeability values 4 orders of magnitude lower than those reported by Rothenberger et al. [23]. In that study, permeability was calculated as the product of solubility and diffusivity coefficients measured in separate experiments. Both solubility and diffusivity measurements were carried out without accounting for the surface state or the presence of oxide layers. Therefore, the permeability values obtained in this way have to be related to “clean” quasi-pristine surfaces.

The discussion of the results of the permeation tests carried out in this work has to take into consideration the analysis of the membrane surface, which, as seen, appears significantly modified after testing due to the presence of oxide layers. As reported in Section 3.1, by increasing the temperature, the nature of the oxide layers changes (from TaO to Ta_2_O_5_ and to atomic O) with an increase in thickness [25,26,27,28].

**Figure 10:**
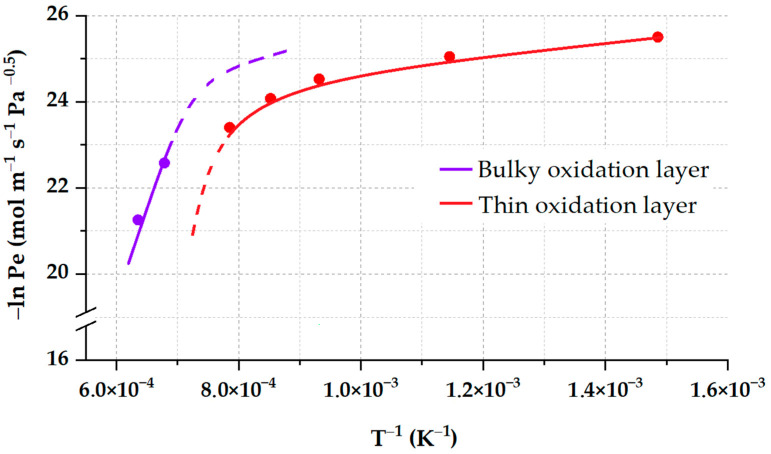
Comparison of hydrogen permeability of Ta covered by thin or bulky oxidation layer vs. temperature.

Depending on their nature and thickness, the oxide layers reduce the permeation flow rate both by reducing the metal surface available to the hydrogen passage and by increasing the contribution to mass transfer resistance due to the surface reactions that could become the controlling step of the hydrogen permeation. Under these conditions, Sieverts’ law, strictly valid for permeation controlled by diffusion bulk mechanisms, should be revised by adopting a coefficient of the partial pressures in Formula (2) ranging from 0.5 to 1. However, taking into account the data set of permeability measurements made available in this experimental campaign, the results analysis has been carried out by applying Sieverts’ law. In this way, at low and high temperatures, different activation energies values have been calculated, corresponding to 2679.8 K (low–medium temperature) and 30,691 K (high temperature), respectively representative of the presence of thin and bulky oxidation layers, as represented in the graph of Figure 10.

## 4. Conclusions

Tantalum tubes have been selected for extracting the hydrogen produced in a membrane reactor exploiting the water-splitting reaction. Thanks to the presence of two membranes selectively permeable to hydrogen and oxygen, the water-splitting reaction occurs with reaction conversions of practical interest at temperatures of and over 1873 K, and the aim of this study was to evaluate hydrogen permeability using tubular tantalum membranes over as wide a temperature range as possible, compatible with the experimental setup. The previous assessments of hydrogen permeability of Ta are limited in practical application since being determined as the product of distinct diffusivity and solubility experiments and then disregarding the effect of oxide layer formation that can introduce significant measurement uncertainties. Further, these permeability data present in the literature reached temperatures up to 1073 K, well below the temperatures of interest for the realization of the water-splitting reaction powered by solar energy. For these reasons, a specific experimental setup has been built for testing commercial tantalum closed-ended tubes under operating conditions that involve the formation of oxidation layers. The permeation tests have been carried out in dead-end mode, and the pressure variations within the membrane lumen have been related to hydrogen permeability using a representative mass-transfer model.

The results showed that the permeation process exhibits two distinct trends in the temperature ranges 673–1273 K and 1473–1573 K, corresponding to significantly different activation energies and pre-exponential factors of the permeability coefficient. Such behavior has been linked to the formation of oxide layers even in the presence of traces of oxygen (a few ppm), as observed with the Ar-H_2_ gas used in the permeation tests. The presence of progressive oxidation of the surface of the tantalum tubes tested at higher temperatures has been verified by SEM-EDS analyses in agreement with the associated reduction of hydrogen permeability.

In future work, some open aspects such the relationship between the oxide layer nature and thickness with the permeation mechanisms should be studied more in detail through additional characterization of the metal surface (e.g., XRD, XPS, Raman spectroscopy, etc.) and a larger statistic of the permeation measurements.

## Figures and Tables

**Table 1:** Pre-exponential factor and activation energy of the hydrogen permeability coefficient.

T, K	Pe_0_, mol m^−1^ s^−1^ Pa^−0.5^	E_a_, K	R^2^	Ref.
673–1273	3.669 × 10^−10^	2679.8	0.902	this work
948–1073	1.0 × 10^−6^	3240	NA	[23]
1473–1573	1.745 × 10^−1^	30,691	-	this work

**Table 2:** EDS quantitative analysis carried out on the cross-section in site 2.

Spectra	O_2_	Cr	Mn	Fe	Ni	Mo	Ta	W	Total (wt. %)
1	8.84						91.16		100.00
2							100.00		100.00
3		11.12	1.28	45.45	6.09		34.32	1.74	100.00
4		17.59	1.41	68.03	10.54	2.43			100.00
5		10.64		44.12	5.69	2.22	34.40	2.93	100.00
6	4.27	7.40		32.84	4.37		51.11		100.00

**Table 3:** EDS analysis of the metal membrane pre-test (*) and post-test (^#^).

Spectra	O_2_	Ta	Total (wt. %)
* 7	2.93	97.07	100.00
	±1.68	±1.68	
^#^ 8	8.03	91.97	100.00
	± 0.34	± 0.34	

## Data Availability

The original contributions presented in this study are included in the article. Further inquiries can be directed to the authors.
